# Adapting the serious illness conversation guide for unhoused older adults: a rapid qualitative study

**DOI:** 10.1186/s12904-024-01485-5

**Published:** 2024-06-17

**Authors:** Abigail Latimer, Natalie D. Pope, Chin-Yen Lin, JungHee Kang, Olivia Sasdi, Jia-Rong Wu, Debra K. Moser, Terry Lennie

**Affiliations:** 1 College of Nursing, University of Kentucky, Lexington, Kentucky USA; 2 College of Social Work, University of Kentucky, Lexington, Kentucky USA; 3 College of Nursing, University of Auburn, Auburn, Alabama USA

**Keywords:** Adaptation, Serious illness conversations, Older adults, Rapid qualitative analysis, Homelessness, Unhoused

## Abstract

**Background:**

Older adults experiencing homelessness (OAEH) age quickly and die earlier than their housed counterparts. Illness-related decisions are best guided by patients’ values, but healthcare and homelessness service providers need support in facilitating these discussions. The Serious Illness Conversation Guide (SICG) is a communication tool to guide discussions but has not yet been adapted for OAEH.

**Methods:**

We aimed to adapt the SICG for use with OAEH by nurses, social workers, and other homelessness service providers. We conducted semi-structured interviews with homelessness service providers and cognitive interviews with OAEH using the SICG. Service providers included nurses, social workers, or others working in homeless settings. OAEH were at least 50 years old and diagnosed with a serious illness. Interviews were conducted and audio recorded in shelters, transitional housing, a hospital, public spaces, and over Zoom. The research team reviewed transcripts, identifying common themes across transcripts and applying analytic notetaking. We summarized transcripts from each participant group, applying rapid qualitative analysis. For OAEH, data that referenced proposed adaptations or feedback about the SICG tool were grouped into two domains: “SICG interpretation” and “SICG feedback”. For providers, we used domains from the Toolkit of Adaptation Approaches: “collaborative working”, “team”, “endorsement”, “materials”, “messages”, and “delivery”. Summaries were grouped into matrices to help visualize themes to inform adaptations. The adapted guide was then reviewed by expert palliative care clinicians for further refinement.

**Results:**

The final sample included 11 OAEH (45% Black, 61 ± 7 years old) and 10 providers (80% White, 8.9 ± years practice). Adaptation themes included changing words and phrases to (1) increase transparency about the purpose of the conversation, (2) promote OAEH autonomy and empowerment, (3) align with nurses’ and social workers’ scope of practice regarding facilitating diagnostic and prognostic awareness, and (4) be sensitive to the realities of fragmented healthcare. Responses also revealed training and implementation considerations.

**Conclusions:**

The adapted SICG is a promising clinical tool to aid in the delivery of serious illness conversations with OAEH. Future research should use this updated guide for implementation planning. Additional adaptations may be dependent on specific settings where the SICG will be delivered.

**Supplementary Information:**

The online version contains supplementary material available at 10.1186/s12904-024-01485-5.

## Background

In the United States of America, in 2023, nearly one in four adults over the age of 55 has experienced homelessness without shelter, and as the population of older adults increases, the number of older adults experiencing homelessness (OAEH) is expected to triple by 2030 [1]. While the impact of COVID-19 and economic recession is still unknown, many OAEH are experiencing homelessness for the first time in late adulthood [1–3]. What is considered “older age” for unhoused populations is unclear, and collecting accurate and timely data on this population can be challenging. However, aging OAEH are more likely to have multiple chronic and life-threatening illnesses sooner, leading to geriatric syndromes in their 50s and 60s [4, 5]. Contending with poor health and multiple chronic conditions [6], the life expectancy of OAEH ranges from 64 to 70 years, considerably less than that of the general population (around 77 years) [7]. Higher rates of mental illness, substance use disorders [8], and victimization [9] in OAEHs further worsen quality of life.

Kelley and Bollens-Lund [10] emphasize that populations with multimorbidity (three or more conditions) represent a subpopulation of unhoused or housing insecure patients with serious illness healthcare utilization, functional impairment, and overall high care needs. Recently, a count of unhoused hospitalized patients in a single night, over half of whom were age 55 and older and had multimorbidities, was estimated to be 20-fold higher than in the community setting [11]. In addition to high rates of multimorbidity for unhoused populations [6], homelessness complicates all aspects of health status [12]. This happens in a variety of ways including homelessness fomented by OAEH physical and mental illnesses (e.g., functional status decline resulting in loss of income or housing), homelessness causing or exacerbating illnesses (e.g., skin disorders, trauma, and malnutrition), and homelessness complicating healthcare treatment (e.g., medication access or proper storage, repeat hospitalizations without outpatient management, and no health insurance) [12]. For example, limited income, food insecurity, and lack of health insurance and assistive devices (e.g., eyeglasses, hearing aids) all worsen health outcomes [13]. Furthermore, OAEH face barriers to accessing healthcare that result in poor healthcare transitions and continuity [14], which are required when managing multiple chronic physical (e.g., hypertension, heart failure, and diabetes) and mental health conditions (e.g., depression, anxiety, and post-traumatic) [4, 6]. While access to health insurance coverage is helpful, the inability to afford co-pays or access to transportation prevents OAEH from having routine clinical care [15], leaving them to navigate life-threatening illnesses on their own. A life-threatening or serious illness is considered “a health condition that carries an elevated risk of mortality and either negatively impacts a person’s daily function or quality of life, or excessively strains their caregivers.” ^10(S-8)^ Currently, there is no agreed upon definition of what constitutes a serious illness for OAEH. However, the harsh environmental conditions OAEH endure while managing, sometimes otherwise treatable illnesses, contribute to the seriousness of their conditions.

## Serious illness conversations for unhoused older adults

Unhoused persons with serious illnesses could benefit from conversations to help them make decisions about their health conditions. Facilitating serious illness conversations reflects a process of understanding what matters most to patients and includes dialogue related to the patient’s knowledge of their health condition and clinical recommendations, prognosis, values, worries, hopes, and goals [16, 17]. These conversations can improve care by reducing the use of life-sustaining therapies at the end of life, increasing awareness of end of life wishes [17] and lowering anxiety and depression [18]. Provider perceptions that death or dying is not a priority for unhoused persons is a barrier to discussing the experience of living with a life-threatening illness [19]. However, unhoused people *want* to talk about their health, especially when thinking about their end-of-life experience or discussing future medical treatment preferences [19–24]. These discussions in unhoused populations have been effective at completing advance directives, naming surrogates, and giving OAEH an opportunity to discuss fears or concerns related to their serious illness [25]. While beneficial, without training and support to facilitate discussions, providers may feel ill-equipped to facilitate these discussions [17]. 

The Serious Illness Conversation Guide (SICG) is an existing tool that offers a script with question prompts, mapping the flow of a serious illness conversation for providers. The purpose of the guide is to facilitate a shared understanding between patient and provider about what is most important regarding the patient’s health and quality of life [26]. It is a tool designed to support professionals who lack serious illness communication training in having serious illness conversations and does not require extensive training or skills. The SICG was initially developed for nurse practitioners and physicians in oncology settings [18], and has been adapted for various settings and populations that include emergency departments with social workers [27], dementia care [28], indigenous populations [29], and telehealth delivery for older adults [30]. However, the guide has not yet been adapted to consider the needs of multiple professions in non-healthcare settings or OAEH.

Guidance and tools are needed to assist nurses, social workers, and other providers who care for OAEH in facilitating serious illness conversations and recognizing and acknowledging the unique needs of this population. Social workers in homeless settings bear witness to tragic and traumatic stories associated with housing instability, oftentimes with little support from supervisors or management, yet they want to reduce clients’ distress [31]. Nurses are also integral in improving health for unhoused populations [32]. A lack of compassionate person-centered care and attention to the unique needs of unhoused people persists [33]. Therefore, our aim in this study was to adapt the SICG for use with OAEH.

## Methods

### Data collection

To adapt the SICG for use with OAEH, we conducted a multi-phase, iterative qualitative study in which we elicited feedback from three groups: OAEH, homelessness service providers, and palliative care providers (as content experts). Data were collected from February through December 2023 in two cities (220,000 and 630,000 population size) in the southeastern United States. We first conducted individual semi-structured interviews with provider experts in homelessness service settings (homelessness service providers) while concurrently completing cognitive individual interviews with OAEH. Analysis occurred during data collection, so once interviews were completed, we modified the SICG based on emergent findings. Lastly, we conducted interviews with expert palliative care providers to make further refinements to the revised (or adapted) version of the SICG. Palliative care aims to alleviate psychological, physical, and spiritual distress for people with life-threatening illnesses [34]. One of the core domains of palliative care is to facilitate medical decision-making and serious illness conversations, making them ideal providers to help further refine the SICG. Interview guides were developed for this study and can be viewed in supplementary files. Our framework to guide adaptations came from Davidson and colleagues’ (2013) Tool Kit of Adaptation Approaches [35]. In their study, they conducted a systematic review of approaches used to adapt interventions, along with 26 interviews from experts in adapting behavior change interventions. They present six typologies of approaches that researchers can consider when adapting interventions, clarifying that not all approaches will be used. These approaches included collaborative working, team, endorsement, materials, messages, and delivery. We focused on a few most relevant to adapting the guide: *collaborative working* (i.e., what is appropriate and effective with target group), *messaging* (i.e., population preferences, resources, and norms), and *delivery* (i.e., preferred communication methods, socioeconomic barriers, and environment). Our process for adapting the SICG is outlined below. Informed consent was obtained by all participants prior to participating in this study. This study was approved by the University of Kentucky Institutional Review Board (IRB # 83,381).

#### Reflexive processes

The research team was intentionally multidisciplinary and comprised of social workers and nurses with clinical experience in homelessness, healthcare, and hospice and palliative care. The primary investigator (AL) of this research is a licensed clinical social worker with over a decade of experience in hospice and palliative care and trained in qualitative research methods. AL and the study’s co-investigator (NP), who has extensive training in qualitative methods and research experience with OAEH, conducted most of the interviews. To promote perspective taking, research team member OS, a registered nurse and doctoral of nursing practice student, also assisted with interviews. Additional team members with varying experiences in nursing, homelessness and palliative care, and the Serious Illness Conversation Guide assisted with transcriptions and templated summaries. The research team met weekly during the study’s 12-month duration to discuss data collection and analysis, keeping a detailed audit trail and memoing reflections. These discussions also allowed team members to reflect on the interview content and their reactions as interviewers and analysts. Palliative care research can be emotionally heavy. Weekly team meetings helped to attend to the well-being of research team members, which promoted their ability to connect with the data [36], collaborate, and work compassionately on this project.

#### Interviews with homelessness service providers

We identified and recruited social work, nursing, and social service providers working with OAEH in homelessness service settings in one state within the southeastern US. Settings included a homeless shelter, a transitional supportive housing site, and a community-based hospital. We conducted two separate interviews with each provider participant using a semi-structured interview format. The first interview focused on understanding existing processes and context to consider when adapting the SICG. The second interview explored providers’ perceptions on adaptations needed to the content of the SICG and implementation at their site. The first interview topics were: (1) individual, organizational, or community-level facilitators and barriers when caring for OAEH and having serious illness conversations, (2) descriptions of any tools or training received related to having serious illness conversations, and (3) examples of when serious illness communication did or did not occur and perceived patient outcomes. The second interview focused on feedback regarding the SICG (e.g., messaging, delivery, collaborative working) and anticipated needs or implementation barriers to using the SICG at their site. Exploring existing processes for facilitating values-based serious illness conversations and reviewing the tool was intended to provide insight and direction into needed adaptations to the SICG and inform training and implementation needs. Interviews were held on site at providers’ service locations, in quiet public places (e.g., coffee shops), or via Zoom. Interviews were audio-recorded and professionally transcribed verbatim.

#### Cognitive interviews with older adults experiencing homelessness

Concurrent with our individual interviews with homelessness service providers, we also separately conducted individual cognitive interviews with OAEH. We worked closely with recruitment site clinicians for referrals and to ensure capacity to consent and participate. recruited OAEH who: (1) were homeless adults aged 50 to 90, (2) had a serious illness, and (3) possessed the capacity to engage in the informed consent process. We operationalized “homelessness” consistent with the Health Resources and Services Administration which considers individuals homeless if they lack housing, reside in public or private facility providing shelter, reside in temporary or permanent housing or other housing programs for homeless [37]. We utilized the serious illness definition as described by Kelley and colleagues (2018) and given the impact of housing, healthcare, and food insecurity on the condition and life expectancy of OAEH, we included a broad range of illnesses such as liver disease, chronic lung disease, diabetes, heart conditions or cardiovascular disease, chronic kidney disease, or cancer.

Our cognitive interviewing process involved individual interviews exploring how OAEH “understand, mentally process, and respond” to the SICG [38]. Although cognitive interviewing is a standard in instrument development, its use among unhoused people or those with cognitive impairments has been limited. However, the method can still be effective and is an important approach for this population [39, 40]. In our interviews with OAEH, we adapted “think aloud” procedures to explain or describe more about how they answered the questions [41]. The think-aloud procedure is one by which participants will verbalize their thoughts about what the questions mean, explaining how they arrived at their answer, any difficulties answering, and other pertinent information they may want to provide [41]. Additional open-ended questions explored participants’ thoughts, feelings, and perspectives about the SICG, and the context of how it might be delivered. Interviews were conducted by a research member trained in having sensitive and difficult conversations with older and seriously ill adults. Interviews were held on sites where OAEH were currently sheltered; they were audio-recorded and professionally transcribed verbatim.

#### Interviews with palliative care providers

Following interviews with homelessness service providers and OAEH (and adaptations made to the SICG), we conducted individual interviews with providers trained in palliative care and currently/ formerly practicing palliative care with OAEH. Interviews included open-ended questions about participants’ roles, scope of practice, and experience in delivering palliative care and facilitating serious illness conversations with OAEH. Participating providers were asked to give feedback and recommendations on how to further modify the adapted SICG. Each interview informed the next where participants were asked to comment on modifications from previous interviews. Interviews were audio-recorded and held either on-site in private settings (e.g., offices) or via Zoom.

### Data analyses

We conducted a rapid qualitative analysis, which is a team-based iterative approach to understanding and exploring complex phenomena from “insiders’ perspectives” and applying knowledge to real-world activities and situations [42]. Rapid qualitative analysis was ideal for this project as it is designed to be used in time-sensitive projects providing timely results and allowing for a “big picture view” of collected data [43–47]. Employing rapid qualitative analysis enabled us to disseminate findings quickly to community partners who facilitated recruitment and expressed a desire in addressing serious illness care for unhoused older adults. Furthermore, findings are comparable with traditional approaches (e.g., thematic analysis), take less time, and are less cost intensive [46, 47]. Methods used in rapid qualitative analysis vary and have been applied in various contexts, particularly in healthcare, to adapt and implement interventions [48–50]. We used two methods to condense the data and identify themes [51], to inform SICG adaptations: templated summaries and matrices analysis. These are described below.

#### Templated summaries

Templated summaries were used as a data reduction technique to promote accessibility and understanding of the data [52, 53]. Templates were organized by a priori categories, or key topics, derived from interview questions. Themes emerged by reviewing transcripts and identifying phrases that reflect surface meanings requiring little to no interpretation by the researcher [52, 53]. We approached our templated summaries like Keniston et al. (2022) using Microsoft Word [49]. Summaries for OAEH reflected key data excerpts related to the SICG’s central domains. In contrast, summaries for homelessness service providers reflected Davidson’s and colleagues’ (2013) Tool Kit of Adaptation Approaches [35] guiding possible areas that may need adaptation (collaborative working, team, endorsement, materials, messages, and delivery). Data segments from the OAEH captured (1) how they *interpreted* specific prompts and questions in the SICG and (2) *feedback* they had on modifications to the SICG. The research team (AL, NP, OS) conceptualized OAEH interpretations of the SICG, based on how they answered the questions and responses from the “think aloud” procedures. Data segments from the homelessness service providers that spoke to their feedback on SICG adaptations were also extracted.

Prior to starting the summaries, the research team worked together to clarify and define each domain of the transcript summaries. This ensured we had a shared understanding of each area (e.g., materials, endorsement, collaborative working) [53]. Each transcript was assigned to a member of the research team; they read and completed an individual summary, pulling data that related to interpretation and feedback on the SICG. Templated summaries were reviewed by AL and NP, doctorly prepared qualitative researchers, to ensure accuracy and consistency with the agreed-upon categories, summaries, and level of interpretation used to reflect the data [52, 53]. 

#### Matrices analysis

Once individual summaries were created, we synthesized this information into three data matrices. Matrices column headings reflected category names used in templated summaries, while row headings were assigned to each participant. Reviewing data segments via a grid enabled the researcher team to compare similarities and differences across transcripts [52–54]. Using matrices allows the researcher to draw conclusions with “immediate, precise, and accessible reference to specific differences of opinion among participant groups”^53(p858)^ The organization of the matrix cells increases the trustworthiness of the data, improving the ability to derive meaning from the data in an organized way [54]. One data matrix focused on SICG interpretation (i.e., how each OAEH interpreted questions of the SICG). The second and third matrix focused on SICG adaptation and collated feedback from the OAEH and homelessness service provider interviews. Lead research team members (AL and NP) reviewed matrices independently, noting patterns and emerging themes. The two team members then met to establish consensus on themes that would inform adaptations. All team members then reviewed themes to establish agreement, a process also taken by Schexnayder et al. (2023) [55]. 

#### Adaptations

The research team adapted the SICG through an iterative process that involved reviewing the data matrices, reading/ reviewing the SICG, and discussing potential changes. We conferenced regularly to discuss questions and issues that came up regarding adapting the guide. Team members worked through places of agreement and difference in our understanding of how OAEH and expert feedback translated into SICG adaptations. A preliminary adapted guide was created for interviews with palliative care providers. To make further refinements, researchers AL and NP made real-time adjustments to the guide during interviews and met collaboratively after every two to three interviews to reach consensus on real-time adaptations that were consistent with adaptations suggested by OAEH and homelessness service providers.

The team included two researchers from social work and two from nursing. Colleagues from the same discipline can often “share the same blind spots” ^(p501)^[56] during analysis (e.g., defining and refining codes); investigator and interdisciplinary triangulation throughout all aspects of the study contributed to the rigor of this project [57]. During all phases of this project (study conceptualization, data collection, adaptation, analysis), we maintained a shared audit trail to document our process, record analytic decisions, and engage with data reflectively [58, 59]. 

## Results

Overall, adaptation themes revealed a need (1) for increased transparency about the purpose and intent of the conversation, (2) to promote OAEH autonomy and empowerment, (3) to align with nurses’ and social workers’ scope of practice regarding facilitating diagnostic and prognostic awareness, and (4) to be sensitive to the realities of fragmented healthcare. Training and implementation considerations also emerged. See Table 1 for data segments from matrices and representative quotes. See Additional files for the adapted guide, The Serious Illness Conversation Guide for Unhoused or Housing Vulnerable Older Adults.

**Table 1:** Adaptation themes reflecting matrices data segments and representative quotes of OAEH (*n* = 11) and homelessness service provider participants (*n* = 10)

Adaptation	OAEH	Homelessness service Providers
Increase transparency about the purpose and intent of the conversation	Response to the question “I would like to talk together about what’s happening with your health and what matters to you. Would this be okay?” OA008 states, “What goes through my head is I’m getting ready to hear some not so good news.”- OA008, 63 year old woman Even if healthcare providers were unsure of the details/ prognosis – he would want to be communicated with. “Let’s do this…lock and load.”- OA010, 70 year old man “Man, don’t fucking lie to me. Keep it fucking real. Don’t let me catch you in some kind of bullshit, man, ‘cause I’m gonna try to catch you in some kinda bullshit, right?” - OA005, 57 year old man “Give it to me straight up. Black coffee.” - OA001, 54 year old man	Need to set up the conversation and communicate why this conversation is important, PROV009, hospital “So, I see myself being a little bit more concise and direct, while also trying to- Look empathetic, and display empathy, and things along those lines…using more succinct, direct comments” that are still “sensitive”- PROV003, hospital
Promote OAEH autonomy and empowerment	Regarding permission seeking, “It’s…like you letting me know that I have a choice. Either I could discuss, or I don’t have to discuss it. Yeah. It gives me that”- OA009, 61 year old man “Just the fact that somebody’s here to help me, you know? To talk to me about it….if they can help somebody else, you know, ease through something, you know? Uh, you know, more power to them, you know? ‘Cause there’s a lot of tough decisions out there, and a lot of them a lot harder than what I’m facing, you know? But of course, I’m dying, but I’m not dead yet. And I’m not on the edge of death, but some of them people got like, days to go or something like that. And I think they’d appreciate some nicer, kind words or something.” - OA001, 54 year old man	Ask for permission to discuss topics (can never ask too many times). Revisit permission. – PROV001, emergency shelter Respect autonomy and ensure they have all the information they may need to make treatment decisions- PROV003, hospital “’Cause then you empower them, letting them share what they know about their illness. And then you get to…ask them… what the doctor has said and to see if they…have heard that conversation or like, if they don’t understand it, that’s another thing that would be good so that, you know, they don’t understand, so let’s get them to talk to their medical professional. So, I think, like, to confirm, like, if they have understanding of their illness, if they need more supports around it. So that would be helpful”- PROV007, transitional supportive housing
Align with nurses’ and social workers’ scope of practice regarding facilitating diagnostic and prognostic awareness	“Uncomfortable…I’m there to see the doctor. Uh, somebody else outside can say, ‘Okay Ed, this, this, this, this and this’ well how do you know? Because you’re not a doctor. A healthcare [worker], I would be more, I have to listen to them. Because…they’re doctors and nurses. And I would listen to them before I would listen to the staff.” – OA003, 63 year old man He feels that if this conversation occurred with one of the shelter staff as opposed to a medical professional, he would question if they really knew what they were talking about regarding his prognosis. “I would think, ‘How do you know that? You’re not a nurse.’ (laughs).”- OA004, 53 year old man	“I try to just always stay away from the medical side to an extent, just because that gets out of my sphere of practice… I think it’s helpful in a way to say like, ‘I can send you in the direction of your provider or whoever that has that information’, because it’s not just coming from me… ‘I’m not a medical provider…not a nurse or doctor…this is outta’ my wheelhouse. I’ll connect you with a doctor but I’m not a doctor.’” PROV007, transitional supportive housing “There’s a lot of protocols, like I… have the authority to…put in orders for labs and medicines, and that kind of stuff… But there is a clear hierarchy and that’s clearly within my protocol, like, I can’t go outside of protocols, and but not, not quite the freedom that I think a palliative care like, navigator, case manager could do.”- PROV004, hospital “But as far as anything in the hospital, like talking about your diagnosis, you know, I can’t really give you any information about that. That’s out of my scope.” - PROV009, hospital
Increase sensitivity to the realities of fragmented healthcare	Lack of permanent housing doesn’t impact her desire to want a provider to talk to her about her health (49:12)- OA007, 72 year old woman Shared frustration about frequent changes in his team while in the hospital and focus on reducing pain medication regardless of his chronic pain. He feels he won’t be remembered and expressed frustration at being told to do things to take care of himself (e.g., go to a clinic), but he doesn’t have resources to do the things he’s being told. He expressed fear about returning to the streets for his safety. -OA001, 54 year old man	May be helpful to print out the conversation guides with their answers stating some men keep a manila folder with important documents to them. – PROV002, emergency shelter RE: the summarizing at the end of the SICG and “…this will help us make sure that your care reflects what’s important to you. How does this seem to you?” – PROV005 feels like this is unhelpful b/c we are often not able to provide the homeless patients with resources. Feels like kind of an empty sentiment.- PROV005, hospital
Training/ Implementation		Time would be a barrier to implementing it…would be interested in training to be able to talk to patients more to help them understand what’s going on from the medical perspective - PROV010, hospital Role, time, and space available are considerations when having conversations- PROV003, hospital “I mean, you have to have someone that’s committed to this, to have a real conversation. I mean, someone that’s really interested in that. And I, I could see it. If they were available the same times you know, and we could just sort of tie the intake with that- um, I think it would have… we could get it done for a lot of people. I just don’t know staff wise who that would be.”- PROV002, emergency shelter When asked about barriers, “In the clinic itself, probably just time barriers, but if we knew, for instance, if it was… If we were participating as part of a project, then that would be somethin’ that would be being important and useful, and we, and we are gaining information from that about not just our patients in working with them directly, but maybe how we manage the entire population. You know, it’s gonna give us good information on what questions. At the end of the day, which of these things have been most important to the people that we’ve talked to in, for focus, you know, in our future management of chronic disease in the houseless population.”- PROV006, transitional supportive housing

### Homelessness service provider interviews

We interviewed 10 providers two times who worked directly with the homeless population across various settings, including the hospital (*n* = 4), ambulatory clinic (*n* = 1), non-profit organization (*n* = 3), and emergency shelter with recovery-based programming (*n* = 2). Most (*n* = 9) of the providers were female and most were non-Hispanic White (*n* = 8); one woman identified as Black non-Hispanic, and one woman identified as biracial. Participants held a variety of job titles (e.g., intake worker, Veterans Affairs coordinator, RN) and represented multiple disciplines (e.g., social work, nursing). Of note, four of the provider participants had previously worked with the unhoused clients/ patients prior to their current employment, with almost nine years of experience on average. See Table 2 for provider characteristics. The first interview ranged from 50 to 94 min, averaging 64 min; the second interview ranged from 15 to 76 min, averaging 38 min.

Many of the same themes in OAEH interviews emerged in our conversations with homeless provider participants. Specifically, providers echoed OAEH participants’ need for more transparency to promote trust and avert paranoia, given the sensitive nature of the questions. Exploring information preferences by asking the question, “How much information about what might be ahead with your health?” felt vague and prompted responses that would be too medically focused for providers’ comfort level. Providers suggested this as an opportunity to elicit information that would help inform referrals or connections to resources, they could make for clients. Based on homelessness service provider interviews, we added language such as, “to make sure I share information that is helpful” and “I’d like you to have the information and support you need” to distinguish support they were able to provide from providing medical advice or suggestions related to improving physical health of OAEH.

Scope of practice concerns guided revisions to the sharing prognosis section of the SICG. Homelessness service providers desired this section to focus on facilitating and comprehending the patient’s understanding of their illness and how much information they had. Practically, most homelessness service providers would not know about the patient’s condition; if they did, they did not feel it was their responsibility to share that information. For example, the nurse and social work participants in community hospitals relayed that it was the attending physician’s responsibility. Therefore, this section was changed to elicit what the patient is “most worried about with their illness” and share a general concern that their “health might get worse” and acknowledge they could get “sicker or injured”. We wanted to include language to reinforce transparency and intent by adding the statement, “to know what’s most important to you if that happens”.

Provider participants felt it important to promote OAEH autonomy and person-centered care throughout the guide by removing “recommendation” language. They also did not feel it appropriate to offer reassurance for continuity of care or that they would receive the best care as they recognized patients’ care experiences were often fragmented. So, we omitted the language at the end of the guide suggesting they will “receive the best care possible” and replaced it with actionable steps that the facilitator would take next.

All provider participants advocated for the needs of OAEH by offering general feedback about the delivery of the conversation. Participants reinforced the need for facilitators to ensure receptiveness, emotional safety, and trust before starting and throughout the conversation. However, some of the suggestions made by provider participants contradicted what the OAEH participants said. For example, several provider participants expressed concern about having serious illness conversations with OAEH, citing concerns that it may be too emotionally difficult for them, or they would not engage willingly. However, this was not the case with our sample of OAEH who expressed a desire to have these conversations. Nevertheless, homelessness service providers also expressed concerns that addressing too many of the emotional aspects of this conversation would be outside of their scope of practice. Therefore, we replaced the facilitator prompt to “validate and explore emotions” with a prompt to “pause and allow silence” with specific language. This finding and other feedback prompted key implementation and training considerations that would need to be considered before facilitating conversations using this guide in any homelessness service setting.

**Table 2:** Homelessness service Provider Participant Characteristics (*n* = 10)

Characteristics	Total
Gender	
Female	9
Male	1
Race	
White	8
Black	1
Bi-Racial	1
Marital Status	
Single	4
Married	2
Divorced	1
Unknown	3
Education Level	
Masters	7
Bachelors	2
Associates	1
Profession	
Social work (Director/Manager/Intake)	6
Nursing (APRN/RN)	3
Non-health related	1
Current Employer	
Community hospital	4
Transitional supportive housing	3
Emergency shelter with RPB	2
Academic medical center	1
Relevant Work Experience	
1 to 5 years	5
More than 5 to 15 years	3
More than 15 to 30 years	2

Note: APRN = Advance practice registered nurse, RN = Registered nurse, RBP = Recovery-based programming

### Interviews with older adults experiencing homelessness

We interviewed 11 OAEH, mostly men (*n* = 9) between the ages of 53 and 72 (*M =* 61). The sample included six white participants and five Black participants. Most were of non-Hispanic descent (*n* = 10). Participants had a range of self-reported illnesses and comorbidities based on past medical diagnoses. These included poor cardiovascular health (e.g., heart failure, hypertension), diabetes, chronic kidney disease, severe or infected wounds, small bowel syndrome, lung disease, and human immunodeficiency virus. All participants reported mental health problems, including depression (*n* = 5), anxiety (*n* = 1), depression and anxiety (*n* = 1), attention-deficit hyperactivity disorder (*n* = 1), post-traumatic stress disorder (*n* = 1), bipolar disorder (*n* = 1), and other non-specified diagnoses (*n* = 1). See Table 3 for characteristics.

Cognitive interviews with OAEH lasted between 53 and 97 min (averaging 70 min); participants answered questions from the SICG and gave direct feedback about their thoughts, feelings, and suggestions regarding the delivery and messaging of the guide’s content. Overall, participants voiced appreciation for having the opportunity to discuss what was important to them regarding their health-related goals, values, and preferences. Changes were made based on participants’ feedback and interpretation of the SICG questions. During the set up and share portion of the guide, participants requested transparency regarding the intent of the conversation. Also, to improve transparency and trust, we included language clarifying the role and discipline of the facilitator and their relationship with healthcare providers. Given that many conversations using the SICG will take place in non-healthcare settings, participants expressed a desire to know what type of healthcare experience the facilitator has (if any) or their relationship with healthcare providers.

None of the OAEH participants wanted to know the prognosis related to “time”; however, they did want direct and compassionate communication about the facilitator’s concerns for their health. Participants’ interpretation of the questions provided insight into how to rephrase questions to elicit responses congruent with the question’s intent. For example, the purpose of the original question, “What would you be willing to go through for the possibility of gaining more time?”, is to explore limit setting regarding invasive treatments (e.g., code status, use of mechanical ventilation) that may go against patients’ values or preferences. However, most participants either did not understand the question or responded with vague or contradictory answers that provided limited detail.

Older unhoused adults endorsed exploratory questions related to their worries, strengths, and activities they enjoy; these questions seemed easy to answer. However, when asked about “the people closest to them”, many found this question to trigger negative feelings (e.g., guilt, shame) because of estranged relationships with relatives and loved ones they were previously close to. Therefore, we removed labeling the relationship with others and asked more neutrally about whether they have “talked about” their worries or what is important to them to “other people”. We then added a follow-up question to identify who they have spoken to as possible health surrogates or collaborators in their care. Closing the conversation also required more clarity. When asked about recommendations, participants typically requested medical information regarding what they needed to do to take care of or improve their health. We revised this question to “is it okay if I share what may be helpful?” to allow more flexibility for providers to provide information aligned with their scope of practice and setting.

Overall, participants requested the conversation be delivered with compassion and respect to ensure they are spoken *with* and not *at*. Participants described past experiences with medical and non-medical providers that influenced their perception and trust of the facilitator. Remaining positive was an aspect of their life that all participants relayed was of critical importance. The difficulty of the conversation did not deter them from having it, but participants did convey that compassion and respect were important aspects to remember when speaking with them. Focusing on the negative or not prioritizing communication that fostered hope was considered scary for their mental health and attitude, given the daily stressors and realities that come with having insecure housing.

**Table 3:** Sample of Older Adults Experiencing Homelessness Characteristics (*n* = 11)

Characteristics	Total
Age in years	M = 60.7 SD= (7.3)
Gender	
Male	9
Female	2
Race	
White	6
Black	5
Marital Status	
Single/Engaged	3
Married/Separated	3
Divorced/Widowed	4
Unknown	1
Education Level	
Doctorate	1
Associates	1
High School/GED	5
9th or 12th grade	3
Unknown	1
Income	
Less than $1,000/month	3
None/Unknown	8
Current Housing	
Emergency Shelter Street Hotel	4
Emergency shelter with RBP	5
Motels/Vehicle	1
Unknown	1
Length of Time without Housing	
1 year or less	3
1.5 to 5 years	3
More than 5.5 years	1
Unspecified/”On and off”	4

Note: M = Average, SD = Standard deviation, GED = General Education Development, RBP = Recovery-based programming

### Palliative care provider interviews

Following preliminary adaptations to the SICG, we interviewed nine providers (two nurse practitioners and seven social workers) with training and past or current experience providing palliative care to OAEH. Their experience reflected work done across the United States in the Southeast (*n* = 6), West (*n* = 1), Southwest (*n* = 1), and Northeast (*n* = 1) across a variety of settings, including inpatient palliative teams within academic medical centers and community hospitals (*n* = 6), a palliative care mobile unit (*n* = 1), an emergency department in an acute care hospital (*n* = 1), and home palliative and hospice care (*n* = 1). Participants had key roles on their respective interdisciplinary teams and settings by engaging unhoused older adults in serious illness conversations, facilitating resources, and coordinating care. See Table 4 for characteristics. Experience in palliative care ranged from 1 year to 30 years, averaging nearly 10 years. Interviews lasted from 36 min to 59 min, averaging 47 min.

Palliative experts were key in further modifying the SICG to reflect core tenets of serious illness conversations. Their knowledge of and skills in advance care planning, goals of care discussions, delivering serious news, and discussing prognosis ensured the spirit of the guide remained intact. For example, experts reinforced the use of phrases and skills such as “I wish, I worry” statements, exploring patients’ understanding of their illness, and seeking permission throughout the guide. Moreover, they aligned these palliative care conversation skills with their knowledge and experience working with OAEH. Sharing worry about the reality that many OAEH may experience acute illness or injury in addition to their chronic and life-threatening illnesses was included in their sharing of concern about their prognosis and increasing transparency about the need for this conversation. Adaptations included language changes that mirror the target populations and decrease power differentials between providers and OAEH. Experts also offered guidance to keep the conversation focused on OAEH’s health, rather than other concerns they may have. The addition of this question, “what are you most worried about with your illness?” was one adaptation made to keep the conversation focused on health. Palliative providers acknowledged that many OAEH have worries that they may bring to the homelessness service provider; without the context of a hospital admission or direct healthcare service provider to guide their thinking, the focus of the conversation may get lost. Additionally, OAEH may have many co-occurring conditions to contend with. This allows the OAEH to identify the illness of most importance/ concern to them and provides insight for the homelessness service provider on what their client may be managing.

**Table 4:** Palliative Care Provider Participant Characteristics (*n* = 9)

Characteristics	Total
Gender	
Female	8
Non-binary	1
Race	
White	9
Education Level	
Masters	9
Profession	
Social work	7
Nursing (APRN)	2
Current Employer	
Inpatient Palliative	6
Palliative Care Mobile Unit	1
Emergency Department	1
Home Hospice	1
Region	
Southeast	6
West	1
Southwest	1
Northeast	1
Years of Palliative Experience	
1–10	7
11–20	0
21–30	2

Note: APRN = Advance practice registered nurse

### Additional takeaways

The following sections outline some central findings that are important to consider when having serious illness conversations with OAEH.

#### Training

All providers interviewed (*n* = 19) suggested aspects that would need to be incorporated into training prior to using the adapted SICG. Homelessness service providers emphasized the need to develop trust and rapport with each OAEH and to recognize the impact of their emotional and mental status on their ability to participate fully in the conversation. For example, many providers discussed the impact of trauma and the need for this conversation to be facilitated in line with trauma-informed care practices. Incorporating those aspects into the SICG training would be needed. How the patient’s symptoms are impacting their life may or may not be a routine part of their role, so training homelessness service providers on how to address this within the flow of the conversation would be helpful. Also, none of the homelessness service providers identified grief and loss training as part of their current roles. While this may be beyond the scope of the training provided before using the guide, this feedback was identified as pertinent to the general care of OAEH and providers, as they care for a population with high mortality. Palliative providers also discussed ways to incorporate serious illness conversation skills into the training portion of the guide. They suggested homelessness service providers may need additional training on what to do if the OAEH declines to answer questions, how to use silence therapeutically, and how to normalize having the conversation.

#### Implementation

Homelessness service providers identified several areas that will need careful implementation mapping and additional adaptation to use the guide appropriately in these settings. Overall, there were considerable differences among providers in the frequency of contacts and time spent with patients based on their setting. For example, there were instances where OAEH were seen repeatedly at an emergency shelter location, but often they were only seen once. Comparatively, in the transitional supportive housing space, OAEH may stay in a space for weeks or months with repeat contact with the social work provider. Providers working at community hospitals would often see OAEH repeatedly but described external pressure to discharge them quickly, thus impacting their ability to engage in lengthier conversations outside the scope of discharge planning. Questions were also raised regarding how long the conversation would take. In addition to having the time available, many homelessness service providers struggled to imagine the timing of and appropriate space to have the conversation. Homelessness service providers expressed concern about whether it would fit within their intake process and wondered whether they were the right person to have the conversation.

Homelessness service providers also questioned the process that would happen after having the conversation. Providers wondered how the information gleaned during the conversation would be used since there is no shared electronic health record system between healthcare and homelessness services. While implementation strategies would address this concern, we removed recommendation language and replaced it with actionable steps (e.g., contacting OAEH provider, completing advance directive). This change puts homelessness service providers in an advocacy and facilitation role to bridge care between homeless and healthcare settings while also reaffirming their commitment to OAEH. Despite the concerns, all homelessness service providers acknowledged the utility and importance of the conversation guide. They were receptive to training and to a tool to help them have a serious illness conversation.

## Discussion

This study is a first step to having nurses, social workers, and other providers use The Serious Illness Conversation Guide for Unhoused or Housing Vulnerable Older Adults in their practice. Our study used an iterative data collection and analytical process of engaging OAEH and homeless and palliative providers to inform adaptations. Adaptations included modifications to increase transparency and sensitivity to the social and emotional aspects of being unhoused. Interviews also reflected the need for implementation planning and training for homelessness service providers before using the SICG in these settings. Limits in scope of practice prompted changes to how OAEH conditions were discussed, while leveraging provider skillsets in facilitation and advocacy. Prognosis adaptations reflect a new tool created by Ariadne Labs (2024) [26], “The Role of Social Workers”. The impact of fragmented and siloed services permeated throughout interviews, both in how OAEH discussed the care they currently received and how providers imagined care they could provide.

Our sample of OAEH was willing and receptive to serious illness conversations and expressed a desire to talk about it with providers. Unhoused people often have previous encounters and experiences with death [21, 60] and have unique fears and worries that they want to be acknowledged [19]. Responding to emotions and speaking with sensitivity and compassion was a priority in our sample and is mirrored in previous research with unhoused populations [19, 20, 23]. Past studies have also shown unhoused people want serious illness communication to be delivered with respect, acceptance, and without judgment [19]. Our participants echoed the need to include language that empowered OAEH and decreased power differentials between patient and provider. While we limited our OAEH to 50 years and older, unhoused adults seem to have chronic illness even younger than “older adult” age; [61] thus, these conversations may need to occur with unhoused persons in their 40s. Overall, using a tool to guide a conversation with OAEH was well received by all participants in our study and emphasizes a needed area of focus in both practice and research.

As stated previously, our approach focused on a few areas offered by Davidson et al. (2013) as guidance for intervention adaptations (i.e., collaborative working, messaging, delivery). Our interviews with the target population (OAEH, homeless and palliative care providers) reflect “exploratory work with the target population…or community leaders” [35] to learn what might be effective and appropriate. The providers we spoke with discussed training needs for utilizing the SICG in non-medical service provision for OAEH. While the homelessness service providers felt ill-equipped to have difficult conversations with clients about serious illness, this is a common experience for many providers when caring for individuals with serious illnesses [62, 63]. Studies suggest that communication training can improve providers’ comfort and self- perceived skills in having serious illness conversations [64, 65]. However, more research is needed to test whether this training improves clinical outcomes as training intervention outcomes are inconsistent [66]. Implementing and evaluating training for homelessness service providers is a needed.

Revisions to the SICG for use with OAEH also reflected messaging adaptations [35]. Individuals experiencing chronic homelessness often have a limited social network with fewer family members and supportive friends, and more ties to individuals in crisis [67, 68]. Original SICG wording such as “how much does your family know about your…wishes” was adapted to remove labeling the relationship with others. Instead, we suggest asking more neutrally about whether they have “talked about” their worries or what is important to them to “other people”. These changes, as well as scope of practice revisions, are examples of messaging adaptations that consider issues unique to the context of OAEH and their non-medical service providers.

Lastly, some adaptations made for The Serious Illness Conversation Guide for Unhoused or Housing Vulnerable Older Adults were related to the delivery of the intervention. Some examples of delivery adaptations include considering the target population’s referred method of communication and addressing potential barriers to participation [35]. In our study, both OAEH and homelessness service providers acknowledged possible emotional barriers that might hinder a fruitful conversation about serious illness. These included a desire for OAEH to maintain positivity, to have direct messaging (to deter paranoia), and for providers to use a person-centered approach. While these considerations are important for serious illness conversations with all patient populations, they are particularly crucial for OAEH. For instance, unhoused adults can experience limited autonomy because of shelter rules and regulations. The connection between trauma and chronic homelessness is also well-established; most people who are homeless have experienced trauma throughout their lifetime [68–71] and homelessness itself is traumatic [72]. While some of our adaptations (e.g., increasing transparency) might encourage psychological safety, we suggest practitioners also couple SICG with a trauma informed approach [73]. 

Our methods present a systematic and timely approach to adapting a communication tool. Like with Adair et al. (2012) [39], using cognitive interviews with unhoused adults proved to be a successful approach to modifying the content and language throughout the guide. Skilled interviewers who took the time to clarify and help participants understand the question were helpful to ensure meaningful responses from the OAEH. In addition to the interviews, using rapid qualitative analysis did not require experience or extensive training from our research team. We completed the study in under 12 months and gathered the data needed to make adaptations, reflecting a timely process. Trustworthiness was upheld by employing multiple strategies to identify patterns in the data, including building consensus via regular team meetings, documenting our process using an audit trail, and using templated summaries and data matrices. Our data has less bias because we did not rely on researchers’ interpretation of the transcripts but on summaries of exactly what the participants said [52]. 

### Limitations

There are additional considerations about our study. First is the nature of the sample. All OAEH participants and homelessness service providers are from one state within the US. While many OAEH shared about life outside the region, most were born and raised there. The sample size of each participant group was small; however, we sought a range of experiences within the group. Yet, aspects of the sample, such as recruitment location, limited the diversity of the sample. The size of the cities where recruitment took place is lower to upper medium density cities and are surrounded by rural counties. Rural homelessness is substantially under-researched but, given geographical barriers, is characterized by unsafe or lack of housing options, jobs, transportation, and healthcare access [74, 75]. The study focused on adapting the guide for those providers who are limited in prognostic delivery, but further adaptations incorporating perspectives of other health and homelessness professionals, such as physicians and chaplains, would be beneficial.

Second, we did not conduct additional modifications after analysis and revising the guide. This study would have been strengthened by gathering perspectives and feedback from participants with the final adapted guide. Interviews with palliative care providers were conducted to offer an iterative approach to adaptations, gradually incorporating modifications with each subsequent interview and building upon the interviews gathered by OAEH and homelessness service providers. Additionally, we were able to use our team’s extensive experience facilitated direction in the adaptation process. All participants were encouraged to contact the research team in between interviews or at the conclusion of their final interview if they had any additional ideas or feedback. We encourage clinicians and researchers to continue to refine the guide and disseminate their methods and results. Next steps for the development of this tool include feasibility and implementation studies, as well as determining the impact training has for homelessness service providers.

## Conclusion

Ultimately, improved housing stability and case management will improve healthcare for OAEH; [76] however, while policy and practice initiatives are developed to address these needs, equipping homelessness service providers with tools to promote serious illness conversations is a promising strategy to improve serious illness care. While training and implementation mapping are needed before initiating this tool in any homelessness service setting, The Serious Illness Conversation Guide for Unhoused or Housing Vulnerable Older Adults paper illustrate a promising first step towards addressing service gaps between healthcare and homelessness services for vulnerable OAEH.

## Electronic supplementary material

Below is the link to the electronic supplementary material.

Supplementary Material 1

Supplementary Material 2

Supplementary Material 3

Supplementary Material 4

## Data Availability

The datasets used and/or analyzed during the current study are available from the corresponding author upon reasonable request.

## Abbreviations

OAEH: Older Adults Experiencing Homelessness
SICG: Serious Illness Conversation Guide
