# Correction: Adapting the serious illness conversation guide for unhoused older adults: a rapid qualitative study

**DOI:** 10.1186/s12904-024-01605-1

**Published:** 2024-12-16

**Authors:** Abigail Latimer, Natalie D. Pope, Chin-Yen Lin, JungHee Kang, Olivia Sasdi, Jia-Rong Wu, Debra K. Moser, Terry Lennie

**Affiliations:** 1 College of Nursing, University of Kentucky, Lexington, KY USA; 2 College of Social Work, University of Kentucky, Lexington, KY USA; 3 College of Nursing, University of Auburn, Auburn, AL USA

**Correction: BMC Palliat Care 23**,** 153 (2024)**

10.1186/s12904-024-01485-5

Following publication of the original article [1], the author reported that the Supplementary file should be updated to include the below information.

This material has been modified by us. The original content can be found at https://www.ariadnelabs.org and is licensed by Ariadne Labs: A Joint Center for Health Systems Innovation at Brigham and Womenʼs Hospital and the Harvard T.H. Chan School of Public Health. Licensed under the Creative Commons Attribution-NonCommercial-ShareAlike 4.0 International License, http://creativecommons.org/licenses/by-nc-sa/4.0/.

The correct Supplementary file is attached here and the original article has been updated.

## Electronic supplementary material

Below is the link to the electronic supplementary material.

Supplementary Material 1
