# Supplementary material for: Adapting the serious illness conversation guide for unhoused older adults: a rapid qualitative study
Source: BMC Palliat Care. 2024 Jun 17;23:153. doi: 10.1186/s12904-024-01485-5 (PMC11181539; doi:10.1186/s12904-024-01485-5)
Supplement: Supplementary file 2 — Supplementary Material 2 [file 12904_2024_1485_MOESM2_ESM.docx]

**INTERVIEW 1**

**Perspectives of individual, organizational, or community-level issues**

**Name:**

**Email:**

**Phone Number:**

**Western Union Card Number:**

**Licensure:**

**Employer:**

**Years in practice:**

**Other relevant working experience:**

Before we start, I’d first like to hear a little bit about the work you do as a nurse/ social worker – in particular working with unhoused older adults who have serious illness.

How long have you been working with this population?

How many clients/patients who are experiencing homelessness do you typically see in a week/ month?

What do you think are some of the challenges facing homeless adults with serious illness?

When you first meet a client who is older (over the age of 50), is experiencing homelessness, and has serious diagnosed medical conditions – what’s your goal in working with them?

- Talk with me about the “best” possible outcome for folks experiencing homelessness along with managing advanced age and health conditions.

What do you see as some of the major challenges in supporting unhoused adults who are also dealing with serious medical issues?

- Thinking on more of an individual level, talk with me about some about what gets in the way of having conversations with unhoused older adults about their serious illness?
- What about your organization/ agency – how does the organization support (or not) you having meaningful conversations with homeless older adults about their care preferences and needs?
- Thinking even broader than individual or organization issues with providing palliative care services to unhoused older adults, can you talk about community level issues that affect your work with this population?
  - Prompts:
    - What resources, like programs, transportation, meals, medical care, etc., are there to support patients once they’re out of the hospital?
      - What is it like to access/ refer patients to them?

**Descriptions of any tools or training received**

I’m curious if there are any tools or resources that you draw on in your work providing serious illness care with this population. Can you talk some about those?

- How did you learn/ first hear about this tool?
- Can you talk about training or support you’ve received in using this tool?
- How does this tool/ resource help in the work you do?
- What are the limitations of this tool, particularly working in this context (setting, population, etc.)?

Can you talk some about any training (either in school or as continuing education) that has been geared towards providing serious illness care to unhoused older adults?

Suppose you were training a new nurse/ social worker to work with homeless older adults experiencing serious illness, what are the important issues they need to know about?

What do you wish you would have known when you first started working with this population?

**Examples of when communication has or has not occurred and perceived patient outcomes**

Can you share with me about instances when you have been able to have a meaningful conversation- or a conversation where you can connect and discuss hopes, fears, and what’s important with an older, unhoused adult about their serious illness and treatment preferences?

- Talk me through that (what prompted it, how did it go, how did the patient seem to respond, etc.)

What about times that you’ve not been able to communicate with patients about dealing with heir serious illness, what gets in the way of you having these conversations?

How does your team support you (or not) in having these conversations?

How does your practice as a <<PROFESSION>> given your scope of practice or licensure impact your initiation of having conversations about serious illness? In other words, do you feel aspects of discussing serious illness such as prognosis or treatment is out of your scope? Or do you not have the licensure or ability to initiate these discussions?

- Discussing their diagnosis/ prognosis?
- Treatment preferences?

How do you think NOT having these conversations with patients affects their health outcomes? How do you think NOT having these conversations with patients might affect their quality of life?

**How would you communicate with residents (shelter staff)**

- Would you? What would you be comfortable discussing
- How would this look if you had training on how to have these conversations

**INTERVIEW 2**

Below is the **Serious Illness Conversation Guide**, which is a tool for talking to patient about their healthcare goals and values. This project is focused on adapting the SICG for use with homeless adults who also have a serious illness.

Given our goal of adaptation of the tool, we would appreciate your feedback on the interview guide, given your work experiences with adults with serious illness and unhoused individuals in particular. Use the comments function in MS Word (Review 🡪 New Comment) to “tag” sections of the interview guide and insert your feedback on the tagged section.

Some suggested things to think about, as you review and comment on the SICG include:

- How you think the question/ phrase might be received by unhoused individuals.
- How you feel (comfort, confidence) about asking the question/ phrase.
- How the question/ phrase might be edited to be more relevant for unhoused individuals.
- Any additions/ edits/ things to consider about language in the conversation guide.
- Any suggestions related to language (clarity, relevance, etc.)
- General thoughts about the usefulness of this conversation guide with unhoused adults; other things we might need to consider in having serious illness conversations with unhoused adults.

| **SICG (Conversation flow)** | **Language used with patients** |
| --- | --- |
| Set up the conversation   - Introduce the idea and benefits - Ask permission | I would like to **talk together** about what’s happening with your health and **what matters to you. Would this be OK?** |
| Assess understanding and preferences | To make sure I share information that is helpful to you, can you tell me **your understanding** of what’s happening with your health now?  How much **information about what might be ahead** with your health would be helpful to discuss today? |
| Share prognosis   - Share prognosis - Frame as a “wish…worry”, “hope…worry” statement - Allow silence, explore emotion | Prognosis:  “Can I share my understanding of what may be ahead with your health?”  *Uncertain*: “It can be difficult to predict what will happen. **I hope you will feel as well as possible** for a long time, and we will work toward that goal. **It’s also possible that you could get sick quickly**, and I think it is important that **we prepare** for that.”  OR  *Time*: “I **wish** this was not the case. I am **worried** that time may be as short as (express a range, ex: days to weeks, weeks to months, months to a year)”  OR  *Function*: “It can be difficult to predict what will happen. **I hope you will feel as well as possible** for a long time, we will work toward that goal. **It’s also possible that it may get harder to do** **things** because of your illness, and I think it’s important that we prepare for that.”  **Pause: *Allow silence. Validate and explore emotions.*** |
| Explore key topics   - Goals - Fears and worries - Sources of strength - Critical abilities - Tradeoffs - Family | “If your health gets worse, what are your **most important goals**?”  “What are your biggest **worries**?”  “What **gives you strength** as you think about the future?”  “What **activities** bring joy and meaning in your life?”  If your illness gets worse, **how much would you be willing to go through** for the possibility of gaining more time?”  “How much do the **people closest to you** know about your priorities and wishes for your care?”  “Having talked about all of this, **what are your hopes** for your health?” |
| Close the conversation   - Summarize - Make a recommendation - Check in with patient - Affirm commitment | “I’m hearing you say that ___ **is really important to you** and that you are **hoping for** ____.  Keeping that in mind, and what we know about your illness, I **recommend** that we ___.  This will help us make sure that your **care reflects what’s important to you. How does this seem to you?”**  “**I will do everything I can** to support you through this and to make sure you get the **best care possible**.” |
