# Supplementary material for: Adapting the serious illness conversation guide for unhoused older adults: a rapid qualitative study
Source: BMC Palliat Care. 2024 Jun 17;23:153. doi: 10.1186/s12904-024-01485-5 (PMC11181539; doi:10.1186/s12904-024-01485-5)
Supplement: Supplementary file 3 — Supplementary Material 3 [file 12904_2024_1485_MOESM3_ESM.docx]

**Feedback on SICG adaptations**

**Name:**

**Address:**

**Email:**

**Phone Number:**

**Licensure:**

**Experience:**

**How many patients do you see/ week-**

**What is the nature of the condition of patients you see**

**What is your primary role**

**What is the transition like with hospice**

**What is your experience as a [discipline] having serious illness discussions**

**How do you navigate scope of practice limitations**

- **EXPLAIN SICG/ SICP**
- **REVIEW CHANGES MADE SINCE LAST INTERVIEW**

IF time:

I’d like to hear from you about potential barriers in implementing the SICG at your agency.

How do you see this tool as being useful (or not) for palliative care nursing/ social work with older adults experiencing homelessness?
