# Supplementary material for: Adapting the serious illness conversation guide for unhoused older adults: a rapid qualitative study
Source: BMC Palliat Care. 2024 Jun 17;23:153. doi: 10.1186/s12904-024-01485-5 (PMC11181539; doi:10.1186/s12904-024-01485-5)
Supplement: Supplementary file 4 — Supplementary Material 4 [file 12904_2024_1485_MOESM4_ESM.docx]

**Interview Guide – Patient Interviews**

*Thank you for agreeing to talk to me! My name is , and I am a researcher at The University’s College of Nursing/ Social Work. We are asking you to volunteer for a research study about how older adults experiencing homelessness cope with serious illnesses and how to talk about them about their illness.* *Doctors, social workers and nurses, as well as facility staff struggle with helping older adults deal with serious illness. We want to give them tools to help that are informed by what’s important to homeless older adults.*

*We are asking you because you are an older adult, are experiencing homelessness, and have a serious illness. We consider a serious illness any kind of health condition that could increase your risk of dying and worsen your daily function or quality of life. One of your doctors, social workers, nurses, or staff person at your facility thought you may be interested in participating.*

*We’re going to ask a few questions to make sure you’re eligible to participate.*

*When was the last time you had permanent or stable housing? (your own place)*

*How long did that last?*

Has a doctor told you that you have a serious health condition?

- 1. Heart condition (heart disease, heart failure, high blood pressure)
     1. Yes
     2. No
  2. Lung disease (COPD, asthma, emphysema)
  3. Cancer (any kind)
     1. Yes
     2. No
  4. Kidney dysfunction (disease, failure)
  5. Diabetes
     1. Yes
     2. No
  6. Infectious disease (HIV/AIDS, TB)
  7. Are there any other health conditions you want to tell us about? [open-ended]

[if not eligible] *I appreciate you taking time to consider the study, but it doesn’t sound like you’ll be eligible for this one.*

[if eligible] *I am going to read over this consent* (hand consent to participant)*, you can follow along, or just listen. I’ll ask you some information about it to make sure you understand it fully. Is that OK?*

(READ CONSENT- USE TEACH BACK)

*Since we are interviewing several people, we will put what everyone tells us together, and no one’s story will be linked to their name. But I am going to record this conversation so I can remember what everyone tells me and put all the information together.*

*I am going to ask for your signature that you are volunteering to be here today and that you understand how I will use the information you tell me.*

*What questions do you have?*

[Collect signed Informed Consent document.]

*First, I am going to ask you a few questions about yourself. You do not have to tell me anything that makes you uncomfortable.*

**Demographic Interview Questions**

1. What is your first name?
2. How old are you?
3. What is your gender?
4. What is your sexual orientation?
5. What is your race? (Circle all that apply)
   1. White
   2. Black or African American
   3. Asian
   4. American Indian or Alaska Native
   5. Native Hawaiian and Other Pacific Islander
   6. Two or more races: _____________________
6. Are you Hispanic?
   1. Yes
   2. No
7. Were you born in the United States?
   1. Yes
   2. No
8. What is the highest level of education you have completed?
   1. 12^th^ grade or less (no diploma)
   2. High School/ GED
   3. Some college, no degree
   4. Associate or technical degree
   5. Bachelor’s college degree
   6. Graduate or Professional Degree
9. Are you currently employed?
   1. Yes
   2. No
   3. [If Yes] Where?
      1. How many hours per week do you work?
10. Have you ever been incarcerated?
    1. Yes
    2. No
11. Are you a Veteran?
    1. Yes
    2. No

12. How would you rate your health?

- 1. Excellent
  2. Good
  3. Fair
  4. Poor

13. Have you been told you have:

1. Arthritis
   1. Yes
   2. No
2. Depression
   1. Yes, how does it present for you?
   2. No
3. Substance Abuse Disorder
   1. Yes
   2. No
4. Obesity
   1. Yes
   2. No

13. How long have you been in this city?

Family history

Have you ever been married?

- 1. Yes, I am now.
  2. Yes, I am not now.
  3. No

Parents: Alive In contact In Location 1 In Location 2

Siblings (ages, gender, geographic location, in contact?)

|  |
| --- |
|  |

Children (ages, gender, geographic location, in contact?)

|  |
| --- |
|  |

Are you in contact with your child(ren)’s mother(s)/ father(s)?

**Cognitive Interview Questions**

A big part of this interview is where I am going to ask you to tell me what you’re thinking about as you answer questions, called thinking aloud. So, before we begin the actual interview, I’d like to ask you a ‘warm-up’ question to introduce you to the think aloud process.

Thinking aloud may be new and unfamiliar to you, but please know there are no wrong answers. I am only interested in knowing what is going through your mind. Any information you provide during this pre-interview will not be used in the project; we want to help you become familiar and comfortable with the ‘think aloud’ process.

Try to visualize the place where you spent yesterday afternoon. Think about your surroundings and what you see in that place. As you recall what you see right around you, tell me what you are seeing and thinking about. (adapted from Willis, 1994)

Now we will be using the **Serious Illness Conversation Guide**, which is a tool for talking to patient about their healthcare goals and values. I’m going to ask you questions about your condition like I am on your treatment team. You may have already been asked these questions or you may be hearing them for the first time. You can answer them honestly and we will relay them to your team if you want us to.

After some of the questions, we will stop so I can ask you about how you understood and responded- that’s the think aloud portion. We just want you to tell us your thoughts on the question itself.

*shaded columns include what we will say in the interviews with patients.

| **SICG (Conversation flow)** | **Language we will use with patient interviews (3/15/23 and after starting using revised SICG)** | **Cognitive interview questions about the SICG language/ patient’s understanding of the language** |
| --- | --- | --- |
| Set up the conversation   - Introduce the idea and benefits - Ask permission | I would like to talk together about what’s happening with your health and what matters to you. Would this be OK? | Can you repeat the question that I just asked you, but in your own words?  What do you think we want to know by asking this question?  If a healthcare provider approaches you with this question, what can they do to make you feel cared for/ OK to talk to them about it? |
| Assess understanding and preferences | To make sure I share information that is helpful to you, can you tell me your understanding of what’s happening with your health now?  How much information about what might be ahead with your health would be helpful to discuss today? | What were you thinking about as you answered these questions?  Can you repeat the question that I just asked you, but in your own words? (Do you remember the question I just asked you about?)  When I asked about “what is likely to be ahead with your illness”, what does that mean to you? |
| Share prognosis   - Share prognosis - Frame as a “wish…worry”, “hope…worry” statement - Allow silence, explore emotion | Prognosis:  “Can I share my understanding of what may be ahead with your health?”  Uncertain: “It can be difficult to predict what will happen. I hope you will feel as well as possible for a long time, and we will work toward that goal. It’s also possible that you could get sick quickly, and I think it is important that we prepare for that.”  OR  Time: “I wish this was not the case. I am worried that time may be as short as (express a range, ex: days to weeks, weeks to months, months to a year)”  OR  Function: “It can be difficult to predict what will happen. I hope you will feel as well as possible for a long time, we will work toward that goal. It’s also possible that it may get harder to do things because of your illness, and I think it’s important that we prepare for that.”  Pause: *Allow silence. Validate and explore emotions.* | How do you remember your medical information?  What do you think about if one of your shelter workers/ staff asked you about your prognosis? Would there be people who you see at the hospital or shelter who you would *not* want to ask you about prognosis?  How do you think about the future when it comes to your prognosis?  How important is it for you to know prognosis? How do you think this would be different if you had stable housing?  Who are the healthcare providers you’ve seen lately about this health issue? Talk with me about the care you’ve been getting recently? |
| Explore key topics   - Goals - Fears and worries - Sources of strength - Critical abilities - Tradeoffs - Family | “If your health gets worse, what are your most important goals?”  “What are your biggest worries?”  “What gives you strength as you think about the future?”  “What activities bring joy and meaning in your life?”  If your illness gets worse, how much would you be willing to go through for the possibility of gaining more time?”  “How much do the people closest to you know about your priorities and wishes for your care?”  “Having talked about all of this, what are your hopes for your health?” | How did you go about answering that question?  Can you share what you are thinking when answering this question?  How easy or difficult did you find this question to answer? Why do you say that?  What does the phrase “abilities so critical to your life” mean to you?  In your own words, how do you understand the idea of “priorities and wishes” with respect to your healthcare?  How is it for you to be asked about the people closest to you vs your family?  What else would you like to be asked about? What else would you think important for your healthcare person or facility staff person to know? |
| Close the conversation   - Summarize - Make a recommendation - Check in with patient - Affirm commitment | “I’m hearing you say that ___ is really important to you and that you are hoping for ____. Keeping that in mind, and what we know about your illness, I recommend that we ___. This will help us make sure that your care reflects what’s important to you. How does this seem to you?”  “I will do everything I can to support you through this and to make sure you get the best care possible.” | What does it mean to have your healthcare person summarize what you talked about with them?  What does the term “treatment plan” mean to you? How does this compare to when we talk about your “care”?  "Would it be OK if we communicated this with your social worker?" |
